# New Elements of Operative Surgery

**Published:** 1847-05

**Authors:** 


ART. III.—*New Elements of Operative Surgery.* By ALF. A. L. M. VELPEAU, translated by Townsend, and with notes and observations by Mott, in three volumes. Vol. 3. Henry G. Langley, Publisher, N. Y. 1847.

Now, at last, we have this great work complete, with a separate volume of plates, and with much valuable matter added by the translator ; and we congratulate the American publishers upon the timely and successful accomplishment of an undertaking so truly formidable. "Authorship," says Schlegel "is, according to the spirit in which it is pursued, an infamy, a pastime, a labor or handicraft, an art, a virtue." To Dr. Townsend, the translator and annotator, this book has been, we think, both a "labor" and a "virtue," attributes which are happily harmonizing and compensating. But we regret that Dr. Townsend should have been so uncourteously and illiberally treated in "sundry *professed* notices and maudlin criticisms" as he chooses to term them in the preface to this volume ; and we beg leave to enter our humble protest against the conduct of the whole army of censors who have assailed by insinuations and broad detractions, this *American* edition of Velpeau's Surgery.

We are happy however to notice that Dr. Mott himself, has in this volume set an example of magnanimity, which, while it is honorable to himself, ought to cover with crimson "those who in their malignity would rob and calumniate both the dead and the living." —(Preface.)

In the second volume of this work, page 650, under the Title, "FIRST AMPUTATION OF THE HIP JOINT IN AMERICA. PERFORMED BY DR. MOTT AT NEW YORK, OCT. 7, 1824," will be seen an excellent wood cut of an old fashioned arm chair, and a kind hearted patient seated upon it, which position he has quietly occupied these twenty years or more, for the sole and most praiseworthy purpose of showing to the public how his stump looked "when perfectly healed;" i. e., as nearly as possible, like a perfectly healed stump in any other part of the thigh. The picture is a good one, and the illustration most satisfactory.

But since this cut was engraved, Dr. Townsend has received evidences from Dr. Walter Brashear, with a letter stating the fact that he had performed the ileo-coxal amputation in August, 1806, at Bardstown, Ky., and the patient recovered. This letter, Dr. Townend, at the request of Dr. Mott, has unhesitatingly published; and in doing so, he very properly calls the attention of the profession to the cheerfulness with which his distinguished relative "abstracts the plume from his own honored brows, that can spare many and much more like this, and affixes it upon the name of the gentleman to whom it rightly belongs." Seldom is the chronicler of just and virtuous actions, called upon to record an act of such noble and disinterested generosity; and we can well appreciate the glow of fervent admiration which induces Dr. Townsend to signalize it by no faint laudation, and to command for it the respectful attention of the world!

This volume comprises what may be called *regional* surgery, including also a very complete section on tumors, and will be considered, we have no doubt, as practically the most important of the three.

*Lymphatic Tumors.*—"Up to the present time, lymphatic ganglions have not been subjected to extirpation but by a very small number of surgeons. The reason of this peculiarity is owing to two circumstances: 1, lymphatic ganglions scarcely ever become engorged or degenerate except from the influence of remote causes; so that there are almost always a certain number of them diseased at the same time, and that it is rarely possible to remove them all ; 2, considered as the result of the disease denominated scrofulous, they have been deemed to form only a symptom, or indication (ombre) of a general affection, so that their removal would remedy nothing, or the least important element only of the malady. In this matter we must understand ourselves correctly. If the lymphatic tumors are in reality imputable to a general constitutional affection, their extirpation should not be attempted. It is the cause which we must first attack and not this feeble symptom. Nor should we moreover extirpate them, when, notwithstanding they have been produced by an external cause, they are numerous and diffused, [disseminees—i. e. existing in various regions. T.] But if there be one only, or if notwithstanding their number, they are well isolated and easy of dissection, we are not to hesitate. If there is reason to suppose the constitution is good, and that the interior of the sphlanchnic cavities is not compromised, their removal offers incontestible advantages. Different also from cancerous tumors, degenerate lymphatic tumors possess also this remarkable feature, that the extirpation of those that are most diseased or most voluminous, rather favors than prevents the diminution (degorgement,) resolution or dispersion of the others. Thus have I frequently confined myself to the extirpation of a single one, or of a certain number of these tumors, though I knew perfectly well beforehand that I should be obliged to leave many others. Having thus extirpated those which were ulcerated, or very salient externally, or those which occasioned most inconvenience and deformity, I have frequently noticed that the others continued in the same condition they were before, or that they afterwards imperceptably disappeared."

With not all of the above are we fully agreed. Some experience in the treatment of lymphatic tumors having convinced us that they will generally be found amenable to well directed and persistent therapeutic means ; and morever that the extirpation of one enlarged lymphatic, where a chain is affected, so far from inducing a subsidence of the remainder, is generally followed by a pretty rapid growth of the others ; this growth generally commences just when the wound produced by the removal of the one has completely closed. If by any chance the wound does not heal kindly, so long as it continues to discharge, the neighboring tumors are held in abeyance. It is thus also, that we have found that while one gland, which had spontaneously suppurated, was discharging, those in the vicinity did not increase. We conclude then, that not only a suppurating gland is a useful drain for the vicious humors contained in other glands, or rather as we should choose to say, an efficient

*counter-irritant*, but also that an enlarged but not softened gland, is in some measure a focus of irritation upon which the irritability of other glands is concentrated and expended. We affirm also that where only one gland is enlarged, its removal is often succeeded by the enlargement of others, and we think that in all cases where we have reason to suspect a scrofulous taint, we ought to be exceedingly slow in concluding to extirpate the tumor.

*Hydrocephalus.*—The question of the propriety of an operation in a case of hydrocephalus occurs so frequently, and so little comparatively has been said or written upon the subject that we have ventured to quote this section entire ; since it contains by far the fullest statistical account of the operation and its results we have yet seen.

"The principal operation which has been proposed for hydrocephalus is puncture of the cranium. Holbrock and Vose (Duges, *Manuel d' Obstetrique*, &c.,) professed to have performed it, or to have seen it performed with success. Rossi (*Medecine Operatoire*, t. II., p. 46,) has drawn in this manner, at several times, six pounds of serosity from the cranium of a child eleven or twelve years of age, and who got well. M. Syme, in 1826, had recourse to it five times on the same child, in the space of a few months, and each time with some apparant advantage, though the little patient ultimately perished. M. Greatwood (*The Lancet*, 1829, vol. II., p. 238,) obtained partial success from it. M. Bedor, (*Gaz. Med.*, 1830, p. 188, who had also made trial of it, likewise believes that it may answer. But the injury done to the brain by hydrocephalus, is ordinarily too deep-seated for a simple puncture in such cases to restore the health. Nevertheless, should it be decided upon, nothing is easier to do than this, either with a lancet, bistoury, or a small trochar.—There would be no other precaution to take than to avoid with care the track of the venous sinuses. Upon the supposition that we did not wish to draw off at once the entire amount of the liquid, I would much prefer repeating the operation from time to time, rather than to leave a canula resting in the wound, as has been proposed by Lecat. As to the rest, it is an operation which now counts a great number of trials. Theodoric (Portal, *Hist. de l' Anat.*, etc., t. I., p. 184,) had already made the remark, that hydrocephalus children treated by the application of the red hot iron to the forehead or the occiput, had ultimately recovered. It appears also that S. Chabbi (Hevin, *Path. Chir.*, t. I., p. 233,) had performed it with success. Also in cases of this disease where it would seem to be required, other surgical means have been resorted to. Warner (*Obs. de Chir.*, obs. 11., p. 69,) says that a case in which extirpation for a *hydrocephalocele* was performed against his advice, it caused death; and Thiebaut (*Journ. de Desault*, t. III., p. 327,) gives the history of two similar attempts, which were followed by the same result. A case also operated upon in Scotland, and in which a hydroencephalocele that projected above the nose was excised, terminated fatally. Leveille (*Nouv. Doct. Chir.*, t. III., pp. 47, 48,) who relates this fact, says the same thing took place at Gottingen. In the case of an infant aged seven months, in which the tumor, projecting through the parietal bone, was cut into by Rambaud, (*Journ. de Dehorne*, t. IV., p. 212,) death in fact followed on the day after the operation. The case of an infant mentioned by Sulpius, (Bonet, *Corps de Med.*, t. IV., p. 6, obs. 7,) was no less unfortunate. I have seen, says M. Champion, two infants die, who were operated upon for this disease in spite of my advice to the contrary, one at the forehead, and the other near the occipital hole. The first died day after the application of the ligature, which had been placed the around the tumor ; the other survived only some hours after the ligature had been applied, followed by excision of a hydrencephalous sac of considerable size.

Puncture of the cranium also for hydrocephalus was performed in France before the English surgeons had received it. Pelletan (Heurtault, *Consider. sur Diff. Points de Chir.*, p. 111, 1811,) had recourse to it at the Hotel-Dieu of Paris, the 7th Thermidor, and year VII, on an infant aged twenty-two months. A canula was left remaining in the parts, and the patient died at the expiration of five days. Besides the above examples, we might mention at the present time many other instances of puncture of the cranium in cases of hydrocephalus. Thus M. Graefe (*Arch. Gen. de Med.*, t. XXVIII., p. 409,) and M. Russel (*Gaz. Med.* 1832, p. 641,) have each had a successful case. M. Hoefeling (*Encyclogz. des Sc. Med.* 1838, p. 251,) gives a case of hydrocephalus in a child aged five years, who having received a kick from a cow, had the cranium fractured and was thus cured of his disease. I will add that M. Allaire, (*Jour. des Conn. Med. Chir.*, t. II., p. 305,) who drew by this operation, repeated three times in one month, six ounces of liquid at each of the two first punctures, and four at the last, had not the same success, as his patient died soon after. It is nevertheless true that the cases of M. Conquest (*Gaz. Med.*, de Paris, 1838, p. 251,) are now sufficiently numerous to merit all the attention of practitioners. In his last table this practitioner relates *nineteen* cases of this operation performed by him during the last ten years. In the first of his cases, M. Conquest, who made but one puncture, drew off 32 ounces of liquid, and obtained complete success. The second underwent three punctures, which yielded thirty-four and a half ounces of serosity, but ultimately ended in death. The third recovered after two punctures and the evacuation of twenty-four ounces of liquid.

In the fourth, death occurred after the fifth puncture and the removal of 48½ ounces of fluid (de matiere.) The fifth died also after four punctures, which furnished 45 ounces of serum ; the sixth was cured by the extraction of 26 ounces of liquid in three punctures; whilst in the 7th, 8th, 9th, and 10th, who died, as well as the 12th and 15th, it is not practicable to make [respectively] but two, one, two, two, one, and four punctures, which obtained 20, 8, 22, 17, 7, and 33 ounces of serosity. The 11th, 13th, 14th, 16th, 17th, 18th, and 19th, which recovered, furnished [respectively] 55, 13, 9, 6, 31, 14, and 9 ounces of liquid, by means of 5, 1, 2, 4, 3, 2 and one puncture for each; from whence we have 9 deaths and 10 cures, on the total amount above mentioned. If it were allowable to count on so so large a proportion of successful cases as this, there could be no doubt that paracentesis of the cranium ought to be practiced in cases of hydrocephalus. But, on one hand, the observations of Pelletan, many similar attempts, collected in the practice of Dupuytren, together with the facts of M. Bedor and M. Allaire, show that up to the present moment, it has scarcely ever succeeded in France. On the other hand, when we reflect upon the possible chances that certain patients might have of living a long time with a hydrocephalus of considerable size, while by puncture they generally succumb at the end of a few days, we have good ground for not deciding upon this operation without some apprehension. In the halls of the Clinque of the Faculty, I have seen a hydrocephalus child of from 5 to 6 years of age, who in other respects appeared to be in sufficiently good health. I have also had an opportunity of seeing a child of from four to five years of age, who had the cranium *quadrupled* in volume, and which a man hawked about the country to exhibit as a curiosity. A hydrocephalus of considerable size, did not prevent a patient who was for a long time seen at the hospital of Perfectonement, from living to the age of 25 or 30 years, and Marechal gives an instance of a patient with hydrocephalus, who attained to his 70th year. These, however, are but rare exceptions, and no one will dispute that hydrocephalus is almost a certain cause of death. A consideration, moreover, which would perhaps influence me in such cases is this, that the existence of hydrocephalic subjects, being accompanied with more or less complete paralysis, and an absolute annihilation almost of the intellectual faculties, is reduced in fact to a vegetative life, and can be of no great moment either to society, the family, or the individual himself; from whence it follows, that without deceiving ourselves as to its importance, we ought, nevertheless, to have recourse to this operation in patients who seem in other respects to be placed under the most favorable conditions possible."

As long ago as June 19th, 1836, the writer made at the urgent solicitation of the parents, the operation of paracentesis capitis, upon a child about one year old, whose head measured before the operation 23½ inches in circumference. The operation and its result is reported in the Feb. No. 1837, of the American Journal of

Medical science. The child survived the operation fourteen days. The operation was made with a small sized trohar. The case was in many points one of unusual interest, and especially from the fact of a hypertrophy of the brain having occurred in one hemisphere and an extensive absorption in the opposite. The inferences which we made from the result and the post mortem examination are thus summed up in the report.

"1st. That the operation hastened the death of the child.

2d. That it was performed under an accumulation of unfavorable circumstances, viz. deficiency of a large portion of cerebral substance, an excessive hypertrophy of other parts, and also an exuberant growth of the cranial bones, their size and perfect union at the base of the skull effectually preventing any considerable degree of approximation, and therefore not affording the brain a sufficient degree of support and compression.

3d. That the result of this case affords no argument against the future performance of the same operation under more favourable circumstances.

4th. That the operation is most likely to succeed when performed early, and that it never can succeed when either of the above conditions exist.

5th. That the water should be discharged gradually, and that the establishment of a *fistulous opening* is feasible and judicious, allowing, when the external pressure is properly applied, a more steady and uniform approximation than can be obtained by any other mode.

6th. That the puncture should be made in the anterior fontanelle, and as far from the mesian line as possible—it being uncertain whether the superior longitudinal sinus holds its accustomed course, or may strike to the one side or the other, as in the above case."

Since then we have repeated the operation upon a child of about the same age, and with a similar result; although the circumstances were apparently more favorable, and we now think we could hardly be induced to resort again to so forlorn a hope.

*Œsophagotomy.*—Though this operation may not have been formally proposed by any person before Verduc and Guattani, it is impossible nevertheless not to perceive that the idea of it is found in several authors who are more ancient. The incision into an abscess containing a small bone which had escaped from the œsophagus and approximated to the integuments of the neck, which had already been performed by Arculanus and Glandrop; and the opening into tumors more or less dense or voluminous, which had become developed upon the same region, as practiced by Kerkring, Rivals, &c., would naturally lead to this operation. But wounds of the œsophagus had, up to that period, been considered so dangerous that practitioners required numerous facts and direct proofs to dissipate their fears and scruples.

This operation was first made by Goursault in 1738, and since by Roland, Arnott, and twice by Begin, making in all *five* cases. An operation so seldom made, and of a character so grave, properly demanded from the author some special notice. He has therefore, devoted to the subject an entire article, and has carefully collected all that is recorded, which was within his reach. It remained still, however, for the American Annotator to supply such information as American surgeons and American Journals might furnish—in short to "do justice to our own countrymen." (Preface.)

We are not a little surprised, therefore, to find that while Dr. Townsend has found room to mention in a note to this article the case of a fish hook lodged in the gullet and removed by Dr. Anthony of Philadelphia, without an operation, he has allowed the only case of the operation of œsophagotomy ever made in America, to be crowded out. We allude to the operation made by John Watson, surgeon to the New York Hospital, upon John Ames, of Massachusetts, and which is reported at length with remarks in the American Journal of Medical Science for Oct. 1844. The case is in many points so unique and remarkable that it could scarcely have failed to have attracted at the time the notice of the whole profession. Mr Ames (his patient) had always had some difficulty in deglutition, but during the year 1843 it became much more serious, and finally resulted in a complete occlusion of the passage. Dr. Watson operated February 12th, 1844, in presence of Drs. Stevens, Rodgers, Hoffman, Post, Buck, and others; making an incision on the left side of the trachea and opening to the seat of the stricture in the upper part of the œsophagus. The stricture was relieved, and a stomach tube carried through the wound into the stomach. On the 6th day the tube was taken from the wound and another introduced through the left nostril and was left undisturbed 25 days. It was then replaced by a fresh tube, which was left in 16 days, when the Dr. was compelled to remove it wholly, owing to a difficulty in respiration etc. In all, the patient had worn the tube nearly 7 weeks. Seven days after, the œsophagus having again become quite closed, the track of the wound, not scarcely healed, was re-opened and a tube carried into the stomach. This was introduced and removed daily, or as he took food, for about a month. The patient was then threatened with suffocation, and Dr. W., assisted by Dr. Buck opened the trachea. Six days after which, and about three months from the time the operation of œsophagotomy was first made, the patient died.—A careful autopsy was made and the case was discovered to possess additional interest from the fact that the stricture was ascertained to be the result of a tuberculous deposit in the tissues.

This operation of œsophagotomy—the only one ever made, so far as the records show, in America, and the only one ever made in any part of the world, so far as we know, for a *stricture* of the œsophagus, certainly ought not to be overlooked in a work which assumes to "do justice to our own countrymen." If Dr. Townsend does not know of it, notorious as the operation was at the time in New York, and recorded as it is in the American Journal of Medical Science, at much length, he certainly is scarcely a proper person to "do justice" to American surgeons. If he did know of it, why was it omitted ? F. H. H.

